# The Predictive Effect of Health Examination in the Incidence of Diabetes Mellitus in Chinese Adults: A Population-Based Cohort Study

**DOI:** 10.1155/2021/3552080

**Published:** 2021-08-11

**Authors:** Xiaomin Fu, Yingmin Jia, Jing Liu, Qinghua Lei, Lele Li, Nan Li, Yanyan Hu, Shanshan Wang, Hongzhou Liu, Shuangtong Yan

**Affiliations:** ^1^Department of Endocrinology, The First Medical Center, Chinese PLA General Hospital, No. 28 Fuxing Road, Haidian District, Beijing 100853, China; ^2^Department of Nephrology, Shunyi Hospital, Beijing Traditional Chinese Medicine Hospital, No. 5 Zhanqian East Street, Shunyi District, Beijing 101300, China; ^3^Clinics of Cadre, Department of Outpatient, The First Medical Center, Chinese PLA General Hospital, No. 28 Fuxing Road, Haidian District, Beijing 100853, China; ^4^Physical Examination Center, Central Hospital of Handan City, No. 59 Congtai North Road, Congtai District, Handan, Hebei Province 056008, China; ^5^Department of Endocrinology, Genetics, Metabolism and Adolescent Medicine, Beijing Children's Hospital, The Capital Medical University, National Center for Children's Health, No. 56 Nan Li Shi Road, West District, Beijing 100045, China; ^6^Department of Endocrinology, The Second Medical Center & National Clinical Research Center for Geriatric Diseases, Chinese PLA General Hospital, No. 28 Fuxing Road, Haidian District, Beijing 100853, China; ^7^Department of Endocrinology, First Hospital of Handan City, No. 25 Congtai Road, Congtai District, Handan, Hebei Province 056002, China

## Abstract

**Background:**

The incidence of diabetes mellitus (DM) was increasing in recent years, and it is important to screen those nondiabetic populations through health examination to detect the potential risk factors for DM. We aimed to find the predictive effect of health examination on DM.

**Methods:**

We used the public database from Rich Healthcare Group of China to evaluate the potential predictive effect of health examination in the onset of DM. The colinear regression was used for estimating the relationship between the dynamics of the health examination index and the incident year of DM. The time-dependent ROC was used to calculate the best cutoff in predicting DM in the follow-up year. The Kaplan-Meier method and Cox regression were used to evaluate the HR of related health examination.

**Results:**

A total of 211,833 participant medical records were included in our study, with 4,172 participants diagnosing as DM in the following years (among 2-7 years). All the initial health examination was significantly different in participants' final diagnosing as DM to those without DM. We found a negative correlation between the incidence of years of DM and the average initial FPG (*r* = −0.1862, *P* < 0.001). Moreover, the initial FPG had a strong predictive effect in predicting the future incidence of DM (AUC = 0.961), and the cutoff was 5.21 mmol/L. Participants with a higher initial FPG (>5.21 mmol/L) had a 2.73-fold chance to develop as DM in follow-up (95%CI = 2.65–2.81, *P* < 0.001).

**Conclusion:**

Initial FPG had a good predictive effect for detecting DM. The FPG should be controlled less than 5.21 mmol/L.

## 1. Introduction

Diabetes mellitus (DM) is caused by various pathogenic factors such as genetic factors, immune dysfunction, microbial infections and their toxins, free radical toxins, and mental factors, leading to hypofunction of pancreatic islets and insulin resistance, which could result in a series of metabolic disorder syndromes, such as electrolytes, and electrolytes are clinically characterized by high blood glucose [1]. In diabetic patients, the proportion of type 2 DM is about 95%, which is more common in middle-aged and elderly people after the age of 30 [2]. In those type 2 DM patients, the secretion of insulin is not low or even higher than the healthy population and the main cause is that the body is not sensitive to insulin, that is, insulin resistance [3].

In recent years, the incidence rate of DM has been increasing. The complications are the biggest cause of death in diabetic patients [4]. Because the cells are incapable to absorb glucose, it remains in the serum. Prolonged high blood glucose can damage the capillaries in the kidneys, heart, eyes, or nervous system, eventually leading to infections, heart diseases, cerebrovascular diseases, renal failure, blindness, lower limb gangrene, and other diseases [5]. The International Diabetes Federation (IDF) estimates that 8.3% of adults (approximately 382 million people) have DM. There are currently 175 million undiagnosed cases, a large amount of whose complications are not noticed [6].

It is not only cost-effective but also a very convenient predicting method to use the health examination indicators of a large population reasonably to provide certain prediction efficiency for potential diabetic patients [7, 8]. Although previous studies reported on the use of health examination indicators to predict DM, most of the models did not analyze the probability of DM during the follow-up period. There was a deviation in estimating the best cutoff [3, 4, 7]. In this study, we aimed to discuss the relationship between the dynamic change of health examination in follow-up years and the incidence of DM. We adopted the time-dependent ROC methods to calculate the best cutoff to discuss the predictive effect of the health examination indicators in the incidence of DM. Therefore, we wished to find the predictive value of health examination in the future incidence of DM.

## 2. Methods

### 2.1. Data Resources

This study was designed based on a population cohort in China. The data were downloaded from the public database which was established by Rich Healthcare Group. The data included the health examination and the incidence of DM which was sorted by Chen et al. [7]. The data included the medical records of the Chinese population from 2010 to 2016. All the participants were at least 20 years old. The inclusion and exclusion criteria were referred from the study of Chen et al. [7]. Briefly, this study included patients with available data of body mass index (BMI) and fasting plasma glucose (FPG) value. All the participants were followed up for at least 2 years. Other health examination indexes included total cholesterol, triglyceride, high-density lipoprotein (HDL), low-density lipoprotein (LDL), alanine aminotransferase (ALT), aspartate aminotransferase (AST), blood urea nitrogen (BUN), and endogenous creatinine clearance rate (CCR). Finally, all the participants with follow-up FPG were included in the study and a total of 211,833 participants were included.

As the acquisition and analysis standard, FPG was collected with at least 10-hour fasting at each visit. The diagnosis standard of DM was defined as FPG > 7.00 mmol/L.

### 2.2. Study Design and Statistical Analysis

This study is aimed at analyzing the predictive effect of the health examination index in the future diagnosis of DM. Firstly, we compared the difference in health examination index between DM patients and those undiagnosed participants. Secondly, we compared the dynamics of health examination based on the visit intervals. Subgroups were divided based on the visit intervals (2-3 years, 3-4 years, 4-5 years, and above 5 years). Next, we tried to find a health examination index to predict the future incidence of DM. We used the colinear regression to find the dynamic change of those indexes based on visit intervals. Due to the incidence of DM collected by follow-up year, we used the time-dependent ROC methods to search for the best cutoff of different health examination indexes. The area under the curve (AUC) was used to estimate the accuracy of the index [9]. Finally, we used the Kaplan-Meier methods to calculate the incidence of DM and used the Cox regression to calculate the HR for incidence of DM and described with 95% confidence intervals (95% CIs). All the statistical significance was defined as *P* value less than 0.05. The data were analyzed by STATA 15.0 (StataCorp, College Station, TX, USA) and R software (version 3.51).

## 3. Results

### 3.1. The Comparison of Health Examination Index between DM Patients and Nondiabetic Participants

As previously mentioned, a total of 211,833 participants were included in our study. During the follow-up years, there were 4,172 participants that were diagnosed with DM in the following year. All the health examination indexes were significantly different (Table 1, all *P* < 0.001). Those patients were older (54.7 years) and have a larger BMI (26.17 kg/m^2^) compared to the nondiabetic cohort (41.8 years and 23.17 kg/m^2^). The initial FPG was higher in the DM group (5.90 mmol/L) compared to nondiabetic participants (4.90 mmol/L). Both cholesterol and triglyceride were greater in DM patients (5.05 mmol/L and 2.09 mmol/L) compared to nondiabetic participants (4.70 mmol/L and 1.32 mmol/L). The trend was also found in AST and ALT (29.1 U/L and 35.2 U/L compared to 23.9 U/L and 23.7 U/L, respectively). Despite the significance shown in statistics, the difference of HDL, LDL, BUN, and CCR between the two groups was not shown. 71.87% of the patients were male, and 4.1% of the diabetic patients had a family history. The percentage of the current smoker was greater in DM cohort patients (35.41%) compared to those without DM (19.74%).

### 3.2. The Comparison of Health Examination Indexes between DM Patients and Nondiabetic Participants in terms of Different Visit Intervals

**Figure 1:**
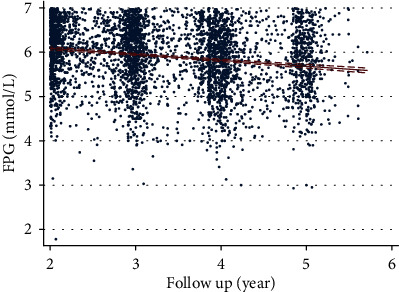
The colinear relationship between initial FPG and the follow-up year in diabetes patients.

Next, we compared the health examination indexes according to different visit intervals (Table 2). The difference was similar to the total cohort. However, we found that the gap of difference was changed dynamically. Also, we found a negative correlation between the incidence of years of DM and the average initial FPG (Figure 1, *r* = −0.1862, *P* < 0.001).

### 3.3. The Best Cutoff and AUC of Health Examination in Predicting Future DM

**Figure 2:**
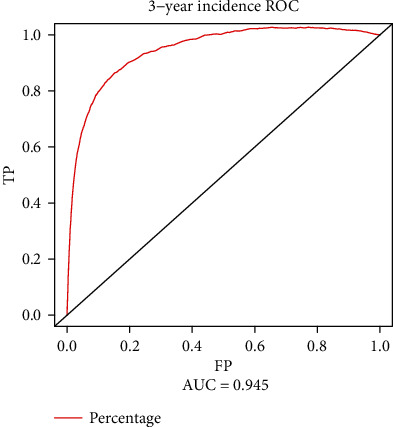
The time-dependent ROC evaluates the best cutoff and AUC of initial FPG (5.2 mmol/L) in predicting the further diagnosis of diabetes.

The best cutoff and AUC were analyzed by time-dependent ROC which had counted the time into the incidence of DM. All the indexes were calculated for the 5-year incidence of DM. The AUCs are shown in Figure 2, Supplement Figure 1, and Table 3. Among all the continuous data, age, BMI, initial FPG, and triglyceride had a good predictive effect in the future incidence of DM (AUC > 0.700). Among these, the initial FPG had a strong predictive effect in predicting the future incidence of DM (AUC = 0.961) with a cutoff of 5.21 mmol/L. For further understanding the predictive effect of initial FPG, we used the index to predict 3-year and 4-year incidence of DM. Both AUCs were larger than 0.94 with a cutoff of 5.49 mmol/L and 5.3 mmol/L, respectively, which means the higher cutoff may have an accurate predictive effect for the shorter period of the incidence of DM (Supplement Figure 2).

### 3.4. The Hazard Ratio for the Incidence of DM

**Figure 3:**
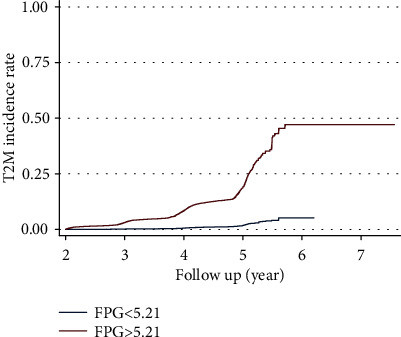
The incident rate of diabetes is based on initial FPG = 5.2 mmol/L.

According to the accurate predictive effect of the health examination indexes reported previously, we used age, BMI, initial FPG, and triglyceride as the factors to calculate the HR for the incidence of DM (Table 4). Due to the better control of the FPG in the population, we adopted the 5.21 mmol/L of FPG as the cutoff for calculating HR. In terms of FPG, we found that the incidence rate of DM was 0.21% in 3 years, 0.67% in 4 years, and 2% in 5 years, if initial FPG was less than 5.21 mmol/L, compared to 3.88%, 10.22%, and 24.35% if FPG was larger than 5.21 mmol/L (Figure 3).

In terms of HR of the DM incidence, participants older than 48 years old had a 1.699-fold chance to have DM (95%CI = 1.635–1.765, *P* < 0.001) compared to younger participants. Participants who have a larger BMI (>24.49 kg/m^2^) may have a higher chance to have DM (HR = 1.499, 95%CI = 1.432–1.566, *P* < 0.001). Similarly, participants who have a higher triglyceride (>1.09 mmol/L) had a higher chance to have DM (HR = 1.48, 95%CI = 1.41–1.56, *P* < 0.001). Most importantly, participants who have a higher initial FPG (>5.21 mmol/L) had a 2.73-fold chance to have DM (95%CI = 2.65–2.81, *P* < 0.001).

## 4. Discussion

In our study, we found that the health examination indexes were significantly different between those patients who would have DM in the follow-up year and those who are nondiabetic participants. We found that the initial FPG in the health examination of healthy participants could have a certain predictive effect on the future incidence of DM. We used the colinear regression method to suggest that the greater initial FPG could predict a shorter incidence of DM and those participants who have an initial FPG of more than 5.2 mmol/L would have a 2.73-fold risk to be diagnosed as DM in the follow-up years.

In the difference of the initial health examination between DM patients and nondiabetic participants, we found that greater age, BMI, initial FPG, cholesterol and triglyceride, AST, and ALT were described in the DM cohort. In terms of BMI and age, Chen et al. had discussed previously [7]. They suggested that young age itself is a remarkable protective factor for developing DM since the prevalence of DM was more common in the middle-aged and elderly population. Several studies showed that BMI was a strong risk associated with the development of metabolic disorders, which includes type 2 DM and cardiovascular diseases [10, 11]. In terms of AST and ALT, which might be related to the liver function, they showed the potential relationship between liver function and the incidence of DM [12]. The liver is the metabolism center of the three major materials of sugar, lipids, and amino acids. It is also an important organ for insulin clearance and the production of inflammatory factors. Insufficiency of insulin secretion and/or function defect characteristic of diabetic patients are mainly manifested as glucose and lipid metabolism disorders [13]. Dysregulation of sugar metabolism may induce hyperglycemia, resulting in the accumulation of glycogen in the liver, thereby causing liver microvascular disease [14]. Lipid metabolism disorder leads to the increased amount of fat that cannot be catabolized and metabolized to accumulate in the liver, forming fatty liver, which impairs liver function. AST and ALT are important indicators that reflect the basic status of liver function, whose changes can sensitively indicate liver cell damage and its degree, as well as liver excretion function [15]. Therefore, continuous monitoring of liver enzyme changes reflects the degree of diabetic liver damage. Oka et al. [16] conducted an epidemiological study on the relationship between elevated liver enzymes and prediabetes. The subjects were 594 patients with normal baseline blood glucose levels, non-B viral hepatitis, or type C Japanese men who are patients or carriers of hepatitis virus. After 3.1 years of follow-up, 141 (23.7%) study subjects progressed to impaired glucose tolerance (IGT), 68 (11.4%) progressed to impaired fasting glucose (IFG), and 23 patients combined IGT and IFG. They also found that elevated ALT may be one of the early changes in the natural course of DM, which not only reflects the state of insulin resistance but also reflects the dysfunction of the gut-insulin axis.

In terms of cholesterol and triglyceride, changes in blood lipid levels in the body cause serious diseases in the body, mainly leading to coronary heart disease and atherosclerosis, and are also related to chronic diseases such as stroke and hypertension [17]. DM patients often have a higher rate of dyslipidemia. The survey results show that the prevalence of dyslipidemia in diabetic populations has reached more than 50% [18]. Although there is no consensus on the mechanism of the mutual influence between blood glucose and blood lipids, researchers in various countries have recognized that there is a certain correlation between blood lipid levels and blood glucose levels [19]. In addition to affecting the prevalence of DM, dyslipidemia is also significantly associated with several complications in diabetic patients. The level of triglycerides also has a significant impact on the development of many complications in diabetic patients. Hypertriglyceridemia can increase the risk of cardiovascular and cerebrovascular remnants in diabetic patients [20]. The transport form of triglyceride in the body is mainly lipoproteins, among which chylomicrons and very-low-density lipoproteins are the main carriers of triglyceride. When diabetic patients have hypertriglyceridemia, the above two lipoproteins can be decomposed into remnant lipoproteins, which accelerate the formation of arteriosclerotic plaques in the body; and as the plaques rupture, platelets accumulate in the body in large numbers, forming thrombi and occluding blood vessels, which ultimately leads to myocardial cell necrosis [21]. Abnormal blood triglycerides also have a certain impact on diabetic nephropathy. Studies have compared blood lipid levels in patients with three types of DM, without nephropathy, early nephropathy, and clinical nephropathy. The results indicate that the occurrence of diabetic nephropathy is related to elevated triglycerides [22].

In the time-dependent ROC, we confirmed that the participants' age, BMI, initial FPG, and triglyceride have higher predictive accuracy in the incidence of DM, in which AUCs were larger than 0.70. Among these, the initial FPG was the significant risk factor associating with the further incidence of DM. Not only in different predictive follow-up years, the FPG had a higher predictive AUC (>0.94), but also, we found that in the Cox regression the initial FPG had a significant impact on the incidence of DM. The incidence rate of DM was 0.21% in 3 years, 0.67% in 4 years, and 2% in 5 years if the initial FPG was less than 5.21 mmol/L, compared to 3.88%, 10.22%, and 24.35% if FPG was larger than 5.21 mmol/L. In 1997, the American Diabetes Association (ADA) and, in 1998, WHO set the critical value of impaired fasting blood glucose as 6.1 mmol/L [23]. Subsequently, in 2003, ADA lowered the threshold to 5.6 mmol/L [24]. In China's 2017 edition of the DM prevention and control guidelines, 6.1 mmol/L ≤ FPG < 7.0 mmol/L is defined as impaired fasting blood glucose [25]. Impaired fasting blood glucose and impaired glucose tolerance are collectively referred to as prediabetes, which are high-risk factors for the onset of DM and can also increase the risk of chronic kidney disease and Alzheimer's disease [26]. During this period, individuals can still be reversible to normal blood glucose. A large prospective cohort study in China shows that daily leisure sports activities (LTPA) are a protective factor for impaired fasting blood glucose and progression to DM, which could reverse the incidence of DM. Reaching the LTPA level recommended by the WHO can effectively reduce the risk of DM (population attributable risk: 19.2%, 95% CI: 5.6%~30.6%) [27].

There were some limitations in our study. Firstly, we only adopted FPG > 7.0 mmol/L as DM, but we did not distinguish the type of DM, including type 1, type 2, and gestational DM. Secondly, we only had the health examination indexes from the database; we did not contain other indexes which may influence the incidence of DM, HbA1c, for example, which might have a higher predictive effect in DM. Finally, we only have an initial experiment, instead of a dynamic test for one participant, which may have a good predictive value for predicting DM.

## 5. Conclusion

In conclusion, we suggested that there were differences in health examinations between participants who had the onset of the DM and those who are nondiabetic participants. Age, BMI, initial FPG, and triglyceride had a better predictive accuracy of DM. Patients who had a higher FPG have a high risk to develop DM; thus, blood glucose should be controlled no matter the circumstance.

## Figures and Tables

**Table 1:** The comparison of physical examination index between diabetes patients and nondiabetic participants.

Variables	Diabetes (*N* = 4,172)	Nondiabetes (*N* = 207,659)	*P*
Age (year)	54.7 (13.20)	41.8 (12.50)	<0.001
Male (%)	3,000 (71.87)	113,123 (54.48)	0.002
Current smoker (%)	415 (35.41)^∗^	11,660 (19.74)^∗^	<0.001
Current drinker (%)	49 (4.18)^∗^	1,302 (2.20)^∗^	<0.001
Family history (%)	171 (4.10)	4173 (2.01)	<0.001
BMI (kg/m^2^)	26.17 (3.48)	23.17 (3.31)	<0.001
Initial FPG (mmol/L)	5.90 (0.71)	4.90 (0.59)	<0.001
Cholesterol (mmol/L)	5.05 (0.94)	4.70 (0.90)	<0.001
Triglyceride (mmol/L)	2.09 (1.50)	1.32 (1.01)	<0.001
HDL (mmol/L)	1.29 (0.34)	1.37 (0.31)	<0.001
LDL (mmol/L)	2.90 (0.70)	2.76 (0.68)	<0.001
ALT (U/L)	26 (18-41)	18 (13-27)	<0.001
AST (U/L)	25 (21-32)	22 (18-26)	<0.001
BUN (mmol/L)	5.01 (1.28)	4.65 (1.18)	<0.001
CCR (*μ*mol/L)	72.7 (15.2)	70.0 (15.8)	<0.001
Final FPG (mmol/L)	7.84 (1.90)	5.08 (0.51)	<0.001

^∗^There were missing data. Age, BMI, FPG, cholesterol, triglyceride, HDL, LDL, BUN, and CCR were described as mean and standard deviation; ALT and AST were described as medians (interquartile ranges (IQR)). Male, current smoker, current drinker, and family history were described as number and percentage. Abbreviation: BMI: body mass index; FPG: fasting plasma glucose; HDL: high-density lipoprotein; LDL: low-density lipoprotein; ALT: alanine aminotransferase; AST: aspartate aminotransferase; BUN: blood urea nitrogen; CCR: endogenous creatinine clearance rate.

**Table 2:** The comparison of physical examination index between diabetes patients and nondiabetic participants in terms of different follow-up years.

Variables	Follow-up 2 to 3 years			Follow-up 3 to 4 years			Follow-up 4 to 5 years			Follow-up above 5 years		
	Diabetes (*N* = 1,764)	Nondiabetes (*N* = 106,055)	*P*	Diabetes (*N* = 1,259)	Nondiabetes (*N* = 57,622)	*P*	Diabetes (*N* = 905)	Nondiabetes (*N* = 35,316)	*P*	Diabetes (*N* = 246)	Nondiabetes (*N* = 8,666)	*P*
Age (year)	54.60 (13.51)	41.71 (12.91)	<0.001	55.00 (13.20)	41.93 (12.56)	<0.001	54.10 (12.60)	42.03 (11.43)	<0.001	52.80 (12.10)	42.06 (11.28)	<0.001
Male (%)	1,257 (71.26)	57,276 (54.01)	<0.001	885 (70.29)	32,106 (55.72)	<0.001	652 (72.04)	18,696 (52.94)	<0.001	206 (83.74)	5,045 (58.22)	0.008
Current smoker (%)	163 (32.34)^∗^	5715 (19.22)^∗^	<0.001	129 (40.20)^∗^	3,526 (21.55)^∗^	<0.001	86 (32.70)^∗^	1,901 (18.57)^∗^	<0.001	37 (44.05)	518 (19.01)	<0.001
Current drinker (%)	23 (4.56)^∗^	728 (2.45)^∗^	0.016	12 (3.74)^∗^	357 (2.18)^∗^	0.042	9 (3.42)^∗^	164 (1.60)^∗^	0.094	5 (5.95)	53 (1.94)	0.054
Family history (%)	57 (3.23)	1799 (1.70)	<0.001	53 (4.21)	1,253 (2.17)	<0.001	50 (5.52)	886 (2.51)	<0.001	11 (4.47)	235 (2.71)	0.079
BMI (kg/m^2^)	26.19 (3.53)	23.24 (3.39)	<0.001	26.11 (3.47)	23.14 (3.31)	<0.001	26.11 (3.45)	23.03 (3.23)	<0.001	26.57 (3.29)	23.18 (3.28)	<0.001
Initial FPG (mmol/L)	6.04 (0.67)	5.00 (0.55)	<0.001	5.86 (0.72)	4.83 (0.60)	<0.001	5.75 (0.74)	4.71 (0.63)	<0.001	5.70 (0.77)	4.73 (0.57)	<0.001
Cholesterol (mmol/L)	5.05 (0.96)	4.70 (0.90)	<0.001	5.05 (0.93)	4.70 (0.90)	<0.001	5.04 (0.94)	4.70 (0.90)	<0.001	5.06 (0.93)	4.69 (0.88)	<0.001
Triglyceride (mmol/L)	2.09 (1.56)	1.34 (1.03)	<0.001	2.08 (1.39)	1.33 (1.01)	<0.001	2.04 (1.44)	1.27 (0.97)	<0.001	2.35 (1.71)	1.31 (1.06)	<0.001
HDL (mmol/L)	1.32 (0.38)	1.41 (0.30)	<0.001	1.31 (0.30)	1.35 (0.31)	<0.001	1.22 (0.29)	1.32 (0.32)	<0.001	1.15 (0.23)	1.32 (0.32)	<0.001
LDL (mmol/L)	2.90 (0.70)	2.77 (0.67)	<0.001	2.91 (0.71)	2.79 (0.69)	<0.001	2.91 (0.71)	2.76 (0.69)	<0.001	2.78 (0.63)	2.63 (0.62)	0.003
ALT (U/L)	25 (18-25)	18 (13-27)	<0.001	26 (18-40)	18 (13-28)	<0.001	28 (18-45)	18 (13-27)	<0.001	29 (20-43)	19 (13-28)	<0.001
AST (U/L)	25 (20-32)	22 (18-26)	<0.001	26 (22-33)	22 (19-27)	<0.001	25 (20-32)	22 (19-26)	<0.001	27 (23-34)	22 (19-26)	0.001
BUN (mmol/L)	5.04 (1.28)	4.66 (1.18)	<0.001	5.02 (1.36)	4.63 (1.18)	<0.001	4.99 (1.23)	4.64 (1.18)	<0.001	4.91 (1.11)	4.69 (1.17)	0.003
CCR (*μ*mol/L)	73.30 (15.90)	70.25 (16.13)	<0.001	72.40 (16.70)	70.06 (15.55)	<0.001	72.20 (15.1)	69.31 (15.37)	<0.001	71.90 (12.1)	69.61 (14.86)	0.01
Final FPG (mmol/L)	7.68 (1.73)	5.06 (0.51)	<0.001	7.87 (1.90)	5.08 (0.51)	<0.001	7.99 (2.04)	5.11 (0.50)	<0.001	8.23 (2.38)	5.08 (0.58)	<0.001

^∗^There were missing data. Age, BMI, FPG, cholesterol, triglyceride, HDL, LDL, BUN, and CCR were described as mean and standard deviation; ALT and AST were described as medians (interquartile ranges (IQR)). Male, current smoker, current drinker, and family history were described as number and percentage. Abbreviation: BMI: body mass index; FPG: fasting plasma glucose; HDL: high-density lipoprotein; LDL: low-density lipoprotein; ALT: alanine aminotransferase; AST: aspartate aminotransferase; BUN: blood urea nitrogen; CCR: endogenous creatinine clearance rate.

**Table 3:** The best cutoff and AUC of physical examination in predicting future diabetes.

Variables	Best cutoff	AUC
Age (years)	48.00	0.73
BMI (kg/m^2^)	24.29	0.74
Initial FPG (mmol/L)	5.21	0.96
Cholesterol (mmol/L)	4.89	0.61
Triglyceride (mmol/L)	1.09	0.73
HDL (mmol/L)	0.51	0.42
LDL (mmol/L)	2.80	0.59
ALT (U/L)	17.30	0.67
AST (U/L)	23.80	0.66
BUN (mmol/L)	4.96	0.58
CCR (*μ*mol/L)	61.90	0.55

**Table 4:** The hazard ratio for calculating the incidence of diabetes.

	HR	95% CI	*P*
Age, >48 years vs. <48 years	1.70	1.63-1.77	<0.001
BMI, >24.49 kg/m^2^ vs. <24.49 kg/m^2^	1.50	1.43-1.57	<0.001
FPG, >5.21 mmol/L vs. <5.21 mmol/L	2.73	2.65-2.81	<0.001
Triglyceride, >1.09 mmol/L vs. <1.09 mmol/L	1.48	1.41-1.56	<0.001

## Data Availability

The data and analytical methods of this study are available from the corresponding author upon reasonable request.

## Supplementary Materials

Supplementary 1 Supplement Figure 1: the time-dependent ROC evaluates the best cutoff and AUC of all physical experiment indexes.

Supplementary 2 Supplement Figure 2: the time-dependent ROC evaluates the best cutoff and AUC of initial FPG (5.3 mmol/L and 5.49 mmol/L) in predicting the further diagnosis of diabetes.
