# Supplementary material for: The Predictive Effect of Health Examination in the Incidence of Diabetes Mellitus in Chinese Adults: A Population-Based Cohort Study
Source: J Diabetes Res. 2021 Aug 11;2021:3552080. doi: 10.1155/2021/3552080 (PMC8377476; doi:10.1155/2021/3552080)
Supplement: Supplementary 1 — Supplement Figure 1: the time-dependent ROC evaluates the best cutoff and AUC of all physical experiment indexes. [file 3552080.f1.pdf]

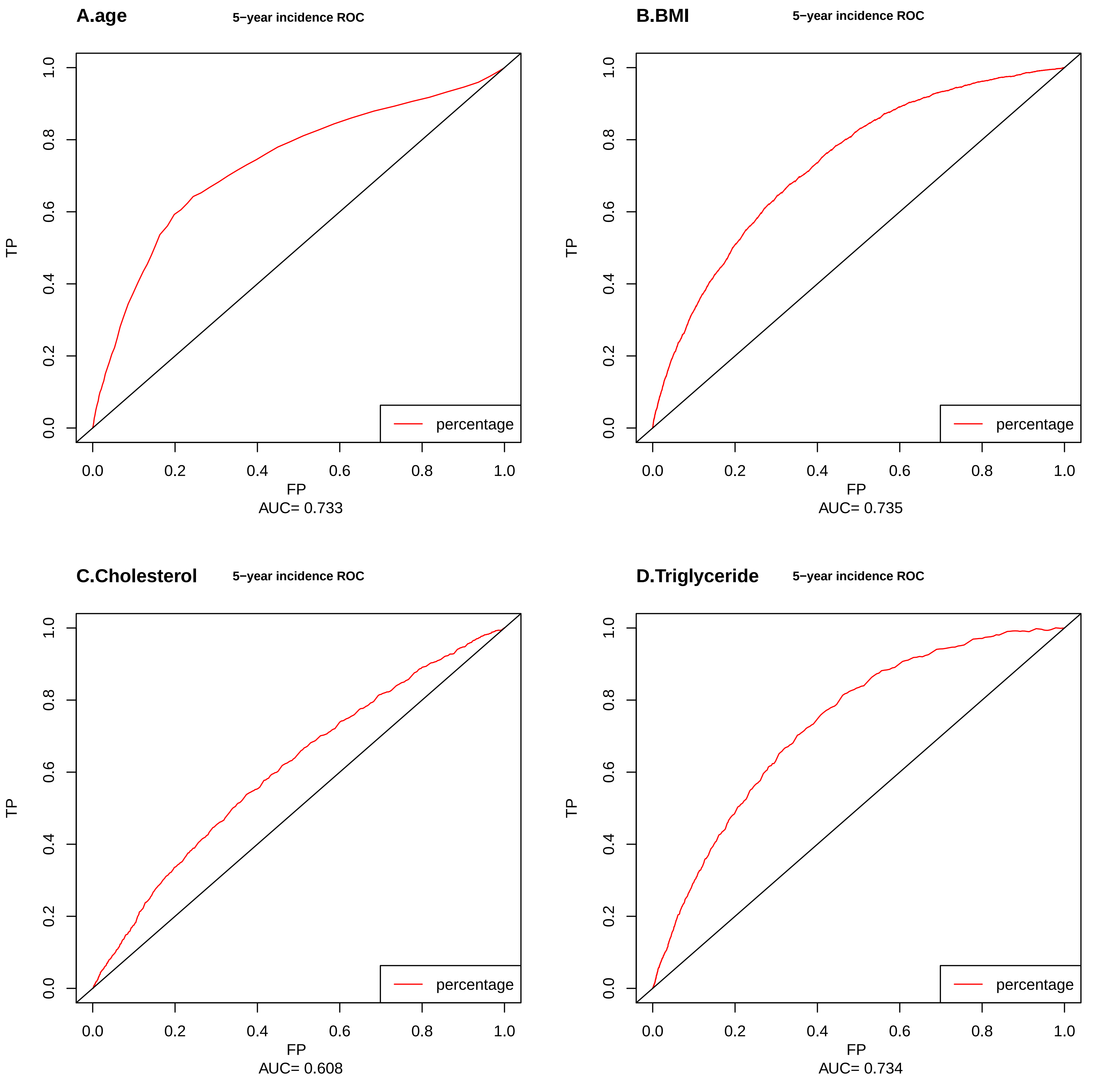
A.age
5−year incidence ROC
TP
FP
percentage
AUC= 0.733
B.BMI
5−year incidence ROC
TP
FP
percentage
AUC= 0.735
C.Cholesterol
5−year incidence ROC
TP
FP
percentage
AUC= 0.608
D.Triglyceride
5−year incidence ROC
TP
FP
percentage
AUC= 0.734

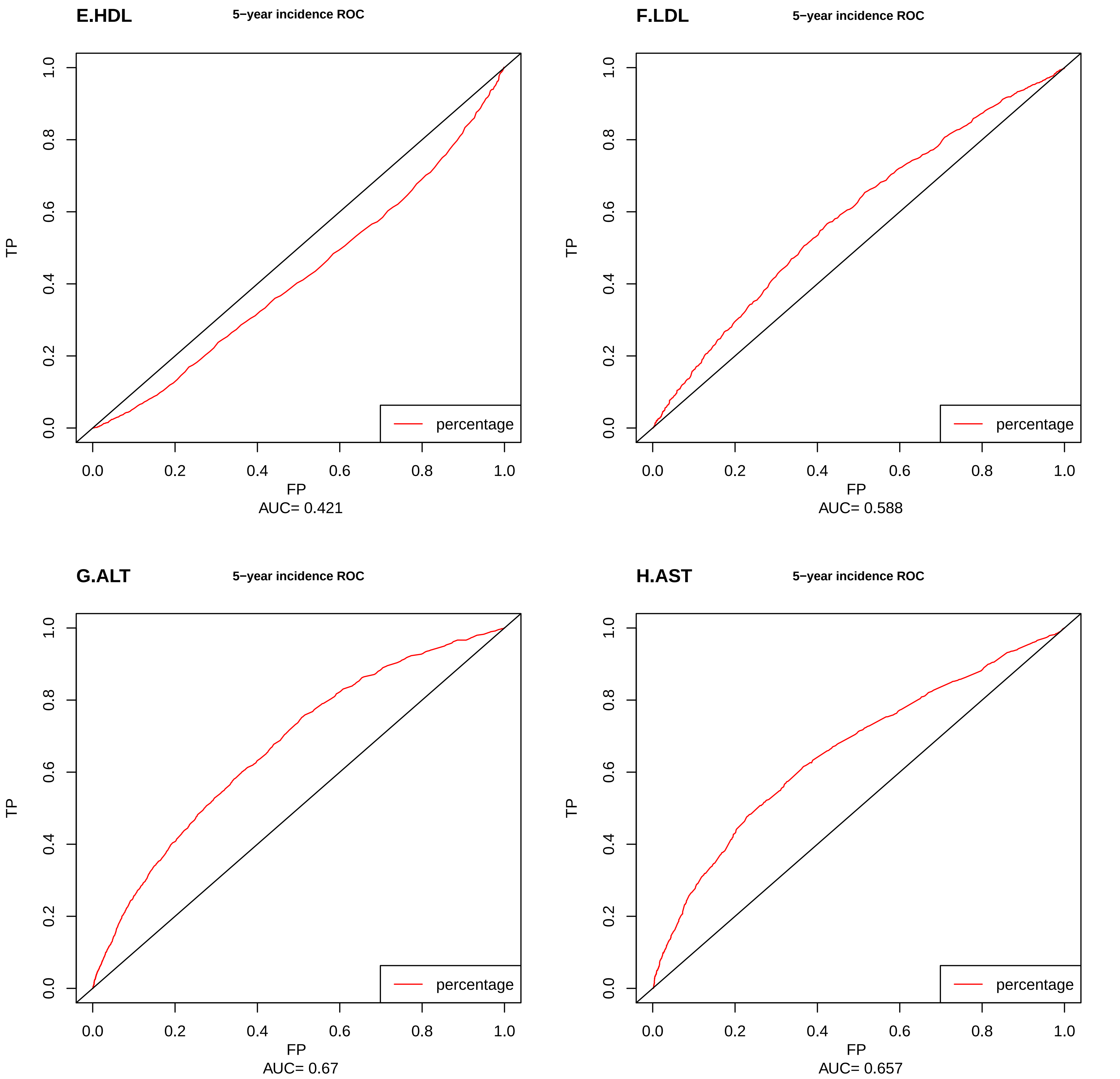
E.HDL
5−year incidence ROC
TP
FP
percentage
AUC= 0.421
F.LDL
5−year incidence ROC
TP
FP
percentage
AUC= 0.588
G.ALT
5−year incidence ROC
TP
FP
percentage
AUC= 0.67
H.AST
5−year incidence ROC
TP
FP
percentage
AUC= 0.657

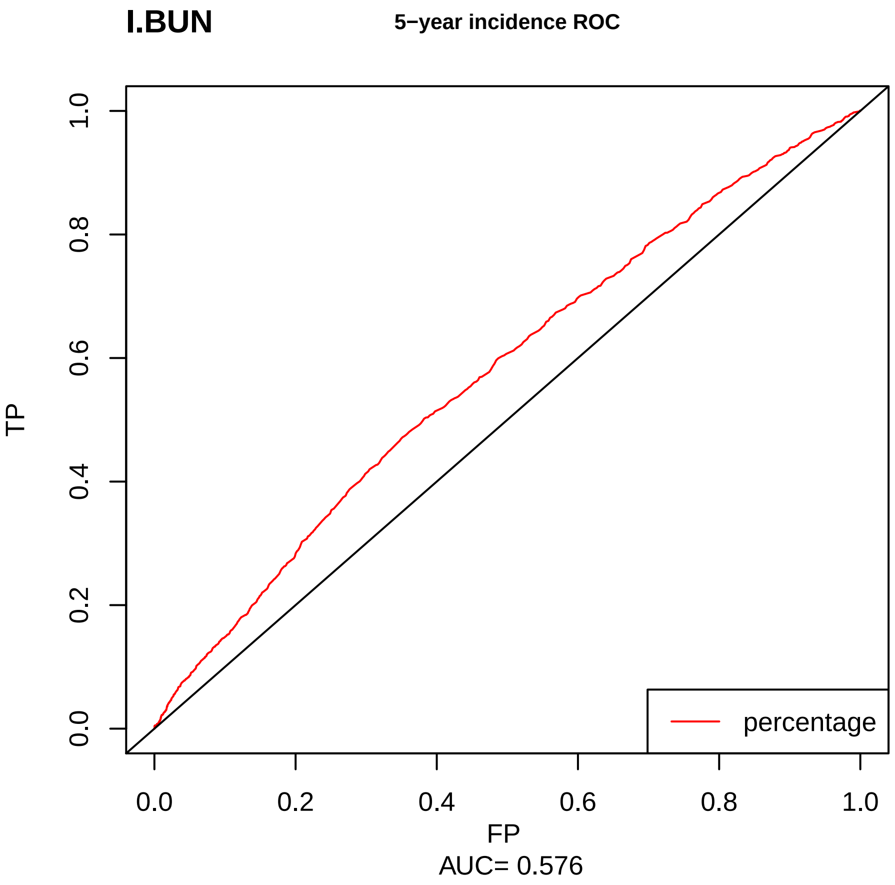

I.BUN
5−year incidence ROC
TP
1.0
0.8
0.6
0.4
0.2
0.0
0.0
0.2
0.4
0.6
0.8
1.0
percentage
FP
AUC= 0.576

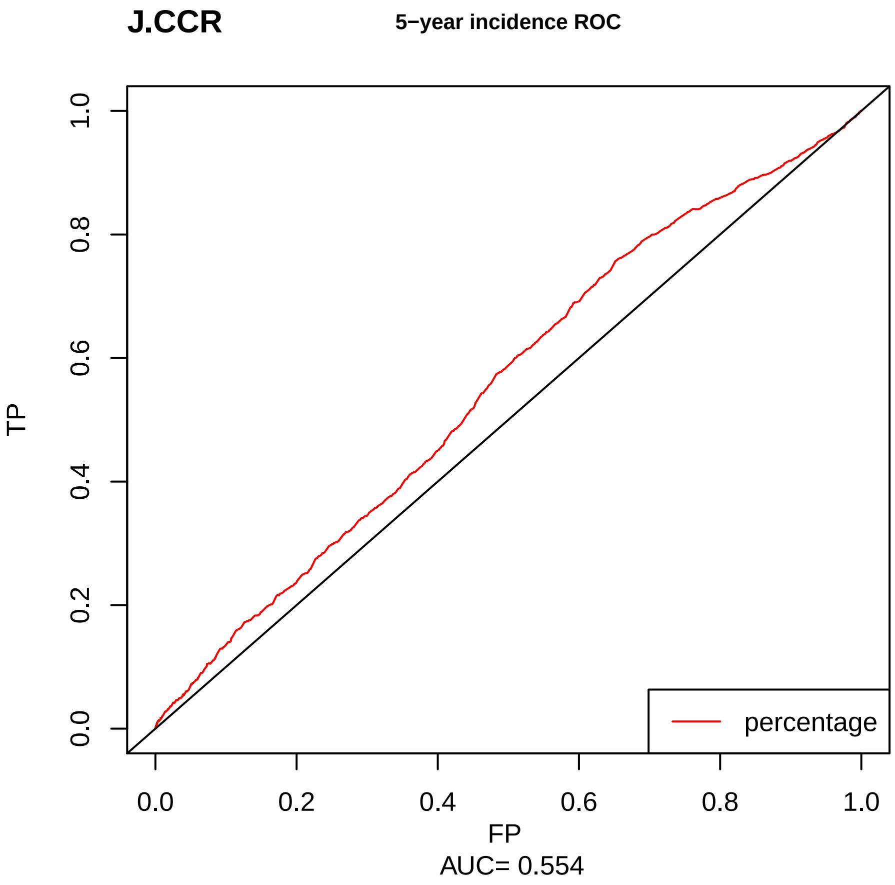

J.CCR
5−year incidence ROC
TP
1.0
0.8
0.6
0.4
0.2
0.0
0.0
0.2
0.4
0.6
0.8
1.0
percentage
FP
AUC= 0.554
