# Supplementary figures and images for: The Predictive Effect of Health Examination in the Incidence of Diabetes Mellitus in Chinese Adults: A Population-Based Cohort Study
Source: J Diabetes Res. 2021 Aug 11;2021:3552080. doi: 10.1155/2021/3552080 (PMC8377476; doi:10.1155/2021/3552080)

**A****3-year incidence ROC**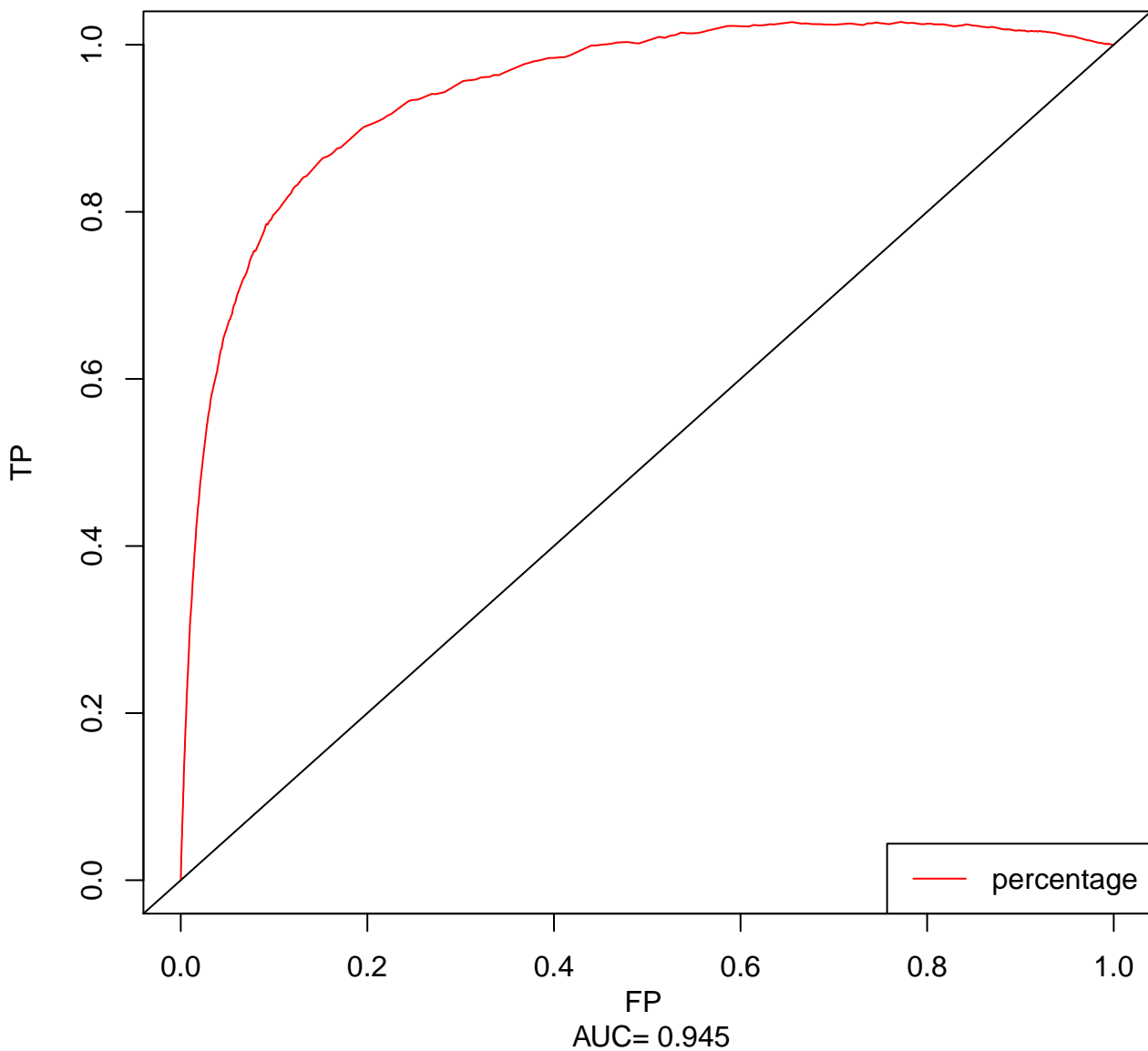**B****4-year incidence ROC**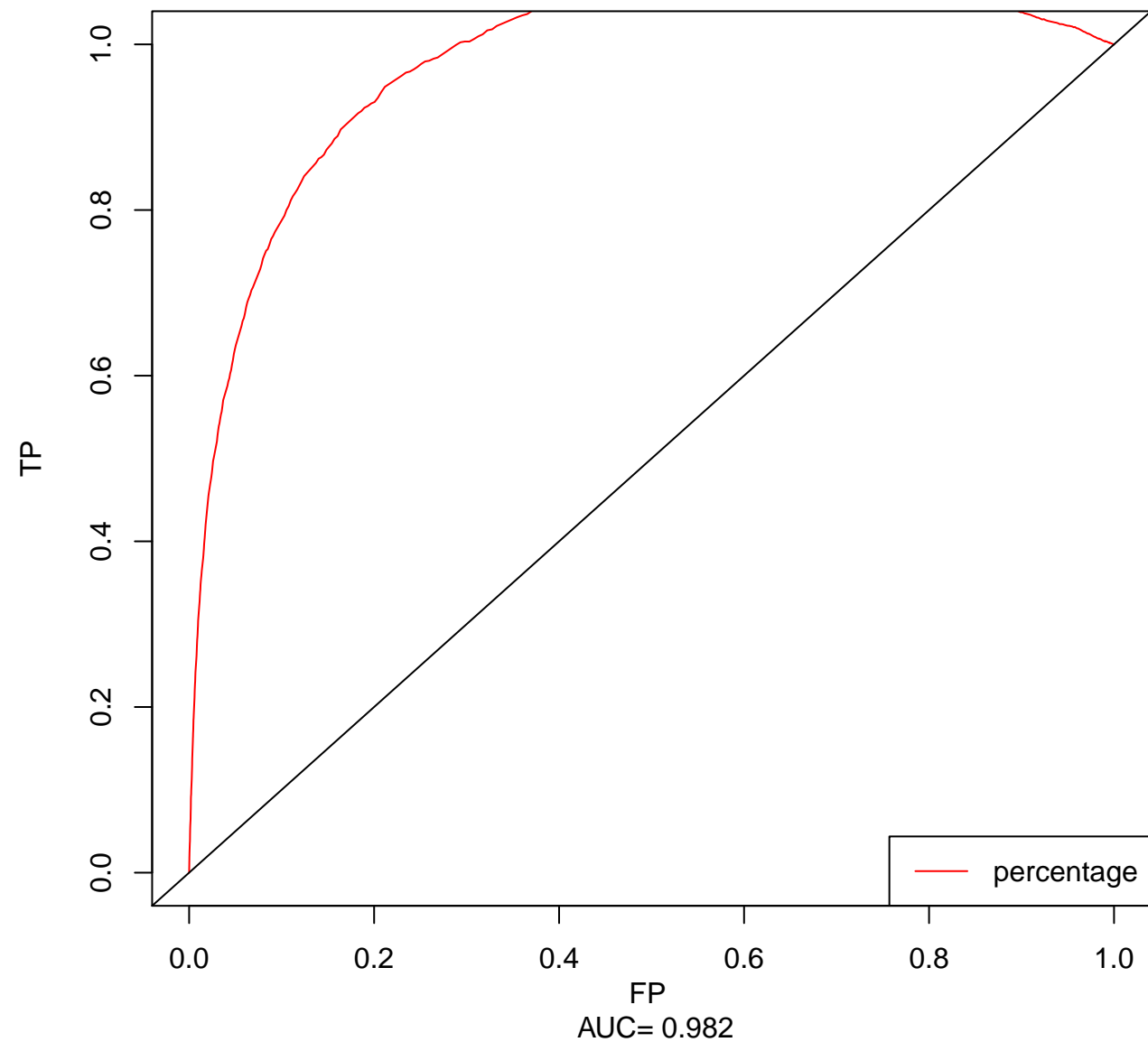

Supplement: Supplementary 2 — Supplement Figure 2: the time-dependent ROC evaluates the best cutoff and AUC of initial FPG (5.3 mmol/L and 5.49 mmol/L) in predicting the further diagnosis of diabetes. [file 3552080.f2.pdf]
